# Abrupt climate shift in the Western Mediterranean Sea

**DOI:** 10.1038/srep23009

**Published:** 2016-03-11

**Authors:** K. Schroeder, J. Chiggiato, H. L. Bryden, M. Borghini, S. Ben Ismail

**Affiliations:** 1 CNR-ISMAR, Venice, La Spezia, Italy; 2 University of Southampton, School of Ocean and Earth Science, Southampton, UK; 3 INSTM, Salamboo, Tunisia

## Abstract

One century of oceanographic measurements has evidenced gradual increases in temperature and salinity of western Mediterranean water masses, even though the vertical stratification has basically remained unchanged. Starting in 2005, the basic structure of the intermediate and deep layers abruptly changed. We report here evidence of reinforced thermohaline variability in the deep western basin with significant dense water formation events producing large amounts of warmer, saltier and denser water masses than ever before. We provide a detailed chronological order to these changes, giving an overview of the new water masses and following their route from the central basin interior to the east (toward the Tyrrhenian) and toward the Atlantic Ocean. As a consequence of this climate shift, new deep waters outflowing through Gibraltar will impact the North Atlantic in terms of salt and heat input. In addition, modifications in the Mediterranean abyssal ecosystems and biogeochemical cycles are to be expected.

The Mediterranean region is recognized as a hot-spot for future climatic changes^1^ and can be described as a “miniature ocean”, since most oceanographic processes of the global ocean occur with a turnover timescale about one tenth of the global ocean’s. This makes it a convenient laboratory, more accessible and controlled because of its semi-enclosed nature and its smaller size, where climate changes happen faster and can be observed on scales of human lifetime. Because of the net evaporation and heat loss, the Mediterranean is an engine transforming fresh and warm Atlantic water (AW) into saltier and cooler Mediterranean waters, which eventually outflow to the Atlantic (Fig. 1). In the northern part of the Western Mediterranean Sea (WMED) open ocean convection occasionally occurs, driven by atmospheric cooling due to strong northerly winds, and by the general cyclonic circulation that uplifts isopycnals in the centre of the gyre^2^, bringing a salty layer of Levantine Intermediate Water (LIW), which comes from the Eastern Mediterranean (EMED), closer to the surface and thus to the influence of the atmospheric forcing. In addition, dense shelf water intermittently forms over the Gulf of Lion’s shelf, and successively cascades down the continental margin, partially contributing to the ventilation of the deep WMED^3^. The water mass that is formed by both processes, deep convection and dense shelf water cascading, is called Western Mediterranean Deep Water (WMDW).

The homogeneous WMDW has long been considered a stable integrating medium to be used to quantify the effects of climate change, by monitoring its gradual potential temperature (θ) and salinity (S) changes^4^. It was known to be characterized by a smooth stratification, observed for decades in the deep WMED interior, with a gradual monotonic decrease of θ and S with depth, at a nearly constant potential density of σ = 29.1 kg m^−3^ (dark grey in Fig. 2A).

## Results

For about half a century the deep WMED heat and salt contents increased almost steadily, with an acceleration after the mid-80 s, ascribed to a variety of forcings as global warming^5^,^6^, increasing temperature and salinity in the inflowing AW^7^, changes in large scale atmospheric patterns^8^,^9^, strait dynamics at Gibraltar and river damming^10^. Since 2005 increases in deep water θ and S two times faster than during 1961–2004 have been reported^11^. Actually, a major deep water formation event in winter 2004/05^12^ set the beginning of the *Western Mediterranean Transition* (WMT^13^). In the following the main features of the WMT are examined: the observed changes in deep stratification, where this study adds new elements to previously known information, and the (east- and westward) propagation of the anomaly within the basin, an aspect which has not been focused on before.

### Changes in deep stratification

The WMT can be defined as a a climate shift which changed the basic structure and properties of the intermediate and deep layers in the WMED: θ, S and σ abruptly increased in the deep layer, and the weak stratification has been replaced by hooks and inversions in the typical θS diagrams at depth (Fig. 2A). Since then this anomaly started to spread from its formation region into the WMED interior, towards Gibraltar and the Tyrrhenian Sea. Different forcings were responsible in triggering the WMT^14^: atmospheric forcings and the advection of anomalously salty and warm LIW due to increased heating and evaporation over the EMED. Such increases have been attributed^15^ to the influence of the *Eastern Mediterranean Transient* (EMT, a climatological event that affected the deep layers of the EMED in the late ‘80 s). The absence of deep convection during the ‘90 s might also have had a role^15^, leading to an accumulation of heat and salt in the intermediate layer. These processes act on different temporal scales, with local air-sea interactions remaining the principal driving force (though modulated by larger scale atmospheric modes^14^) for the Mediterranean circulation. However, the occurrence of extreme or abrupt events like the WMT would depend on the contemporary presence of the appropriate oceanic conditions^16^, a consequence of processes acting on the interannual/interdecadal scale.

**Figure 1:**
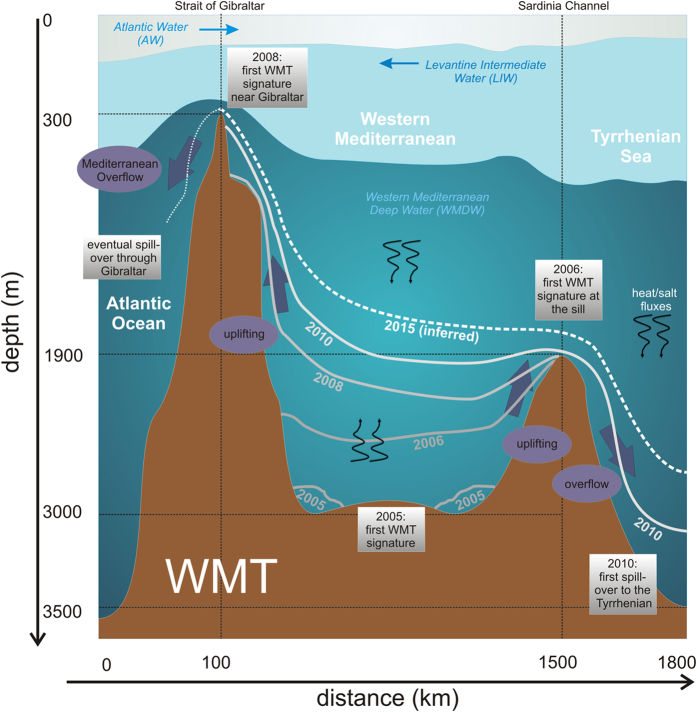
Temporal and geographical evolution of the processes associated to the WMT. The lines denoted by the years indicate the upper interface of the new WMDW. For 2015 a mean uplifting of this interface has been inferred from single stations (shown in Fig. 2A,C), rather than from a whole transect. The dotted line in the Atlantic is a speculation about an eventual spill-over through Gibraltar, which will happen if the process continues.

**Figure 2:**
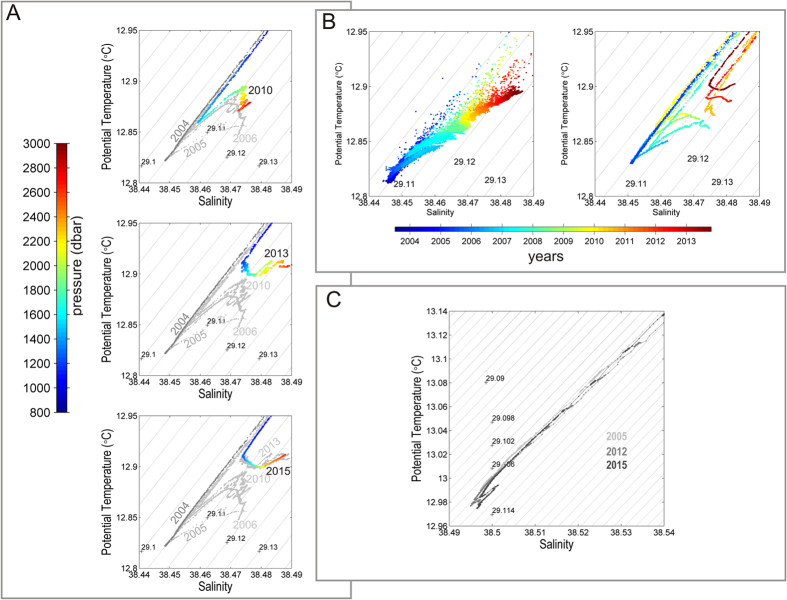
(**A**) θS diagrams from 800 m depth to the bottom, in a repeat station (2800 m, 37.98 °N, 4.65 °E, westernmost red diamond in Fig. 3) of the southern WMED (pressure is colour coded) in 2010 (upper panel, light grey points refer to preceding years, i.e. 2005–2009, dark grey points refer to 2004, the pre-WMT situation), 2013 (middle panel, light grey 2005–2010, dark grey 2004) and 2015 (lower panel, light grey 2005–2014, dark grey 2004); (**B**) θS diagrams at the sill in the Sardinia Channel (1900 m, 38.33 °N, 9.33 °E), (left panel) from bottom mooring data and (right panel) from repeated CTD profiles (years are colour-coded); and (**C**) θS diagrams in a repeat station (3500 m, 38.92 °N, 13.3 °E, easternmost red diamond in Fig. 3) of the southern Tyrrhenian Sea in 2005 (pre-WMT situation), 2012 and 2015.

**Figure 3:**
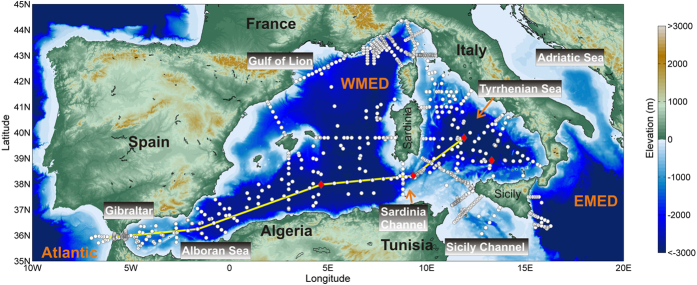
Station map of 30 cruises (carried out by the Italian National Research Council, CNR, on board of R/V URANIA and R/V MINERVAUNO) between 2004 and 2015 (^#^Stations > 1600). Red diamonds highlight stations that are discussed in the text and plotted in Figs 2 and 4, the yellow line connecting the Strait of Gibraltar and the Tyrrhenian Sea is the transect displayed in the schematics of Fig. 1 (map generated by using MATLAB 7.1 http://uk.mathworks.com/products/matlab).

Physical data gathered between 2004 and 2015 during 30 oceanographic cruises (Fig. 3) revealed a decade of enhanced thermohaline variability in the deep WMED. The temporal and spatial evolution of the WMT is outlined in Fig. 1, a sketch of a vertical west-east transect crossing the Strait of Gibraltar, the southern part of the WMED, the Sardinia Channel and the southern Tyrrhenian Sea (for geographical names refer to Fig. 3). After the major event in 2004/05, also in winter 2005/06 large volumes of anomalous WMDW formed, while the following winters 2006/07 and 2007/08 were relatively mild and no deep convection occurred^17^. Dense waters started to be produced again in winter 2008/09 as well as in the following winters (i.e. 2009/10, 2010/11 and 2011/12^18^,^19^). In particular, winter 2011/12 was exceptionally cold over Europe and the Mediterranean^20^. Each winter a new warmer, saltier and denser deep water is formed, leading to a stepwise increase of heat and salt contents in the deep layer and the deep θS diagrams became increasingly complex (Fig. 2A).

**Figure 4:**
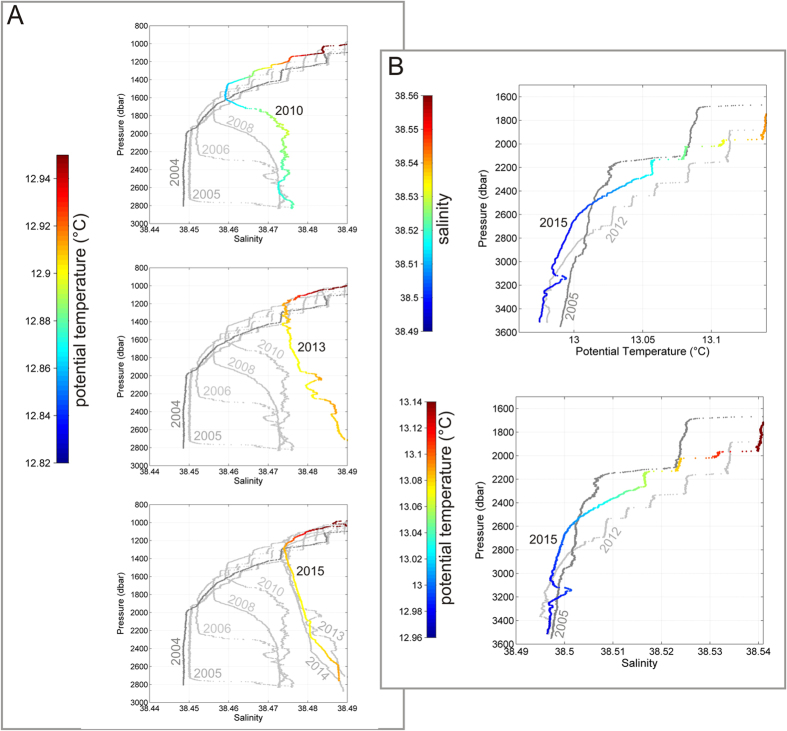
With the aim to highlight the changes of the vertical structure of the water column, here we show (**A**) same data as in Fig. 2A, but in form of vertical profiles (>800 dbar) of salinity (potential temperature is colour-coded); and (**B**) same data as in Fig. 2C, but as vertical profiles (>1500 dbar) of potential temperature (upper panel, salinity is colour-coded for 2015, light grey points refer to 2012, dark grey points refer to 2005, the pre-WMT situation) and salinity (lower panel, potential temperature is colour-coded for 2015, light grey 2012, dark grey 2005).

The new deep waters do exhibit such different properties than the old one that they can straightforwardly be identified in deep CTD casts: a natural tracer in the form of warmer, saltier and denser deep water. Over time the signature became visible in wider and wider regions, allowing a time-scale estimate of the spreading, which could only have been possible with a designed tracer release experiment. Schroeder and colleagues^21^ have followed the propagation of the anomaly throughout the WMED from 2004 to 2006. Here we continue the chronological description of the uplifting old deep water, replaced near the bottom by the new ones (Figs 1, 2A and 4): a near-bottom salty and warm vein intrudes in 2005, and this layer has become 600 m thick in 2006, almost 1000 m thick in 2008, more than 1200 m in 2010, 1400 m in 2013 and >1500 m in 2015.

### Eastward propagation of the anomaly

The deep isopycnals delimiting the interface between new and old deep waters show a doming both towards the Sardinia Channel, to the east, and the Strait of Gibraltar, to the west (Fig. 1). The Sardinia Channel (sill at 1930 m) allows exchanges of the upper part of the deep waters to occur between the WMED interior and the Tyrrhenian Sea. At the sill a CTD station has been performed at least once a year (Fig. 3, second red diamond from west) and a deep sea mooring (belonging to the CIESM HYDROCHANGES Programme^18^) monitors the overflowing dense waters in detail. The densest part of WMDW, trapped in the deep WMED interior, is overflowing the sill when uplifted by even denser WMDW. While until 2005 only the “classical” old WMDW was found at the sill, the new denser WMDW started to cross it since then (Fig. 2B), with the interface between old and new deep water becoming about 200 m shallower here between 2006 and 2009. By 2014 the whole layer below the LIW (>500 m, i.e. the halocline/thermocline and the deep water) has densified to values of 29.11–29.12 kg m^−3^, becoming denser then the “classical” resident water found at <3000 m in the Tyrrhenian Sea^22^. In 2009 the new dense water was firstly detected along the axis of the Sardinia Channel (not shown) on the way to the Tyrrhenian, while in 2010 first signals (not shown) were evident in the interior of the Tyrrhenian (at 39.8 °N 11.9 °E, second red diamonds from east in Fig. 3). In 2012–2015 the thickness of the modified deep layer increased to almost 1000 m (Figure S2b) and the signature of the WMT in the Tyrrhenian Sea (typical hooks in the θS diagram, Fig. 2C) was clear in almost all stations in the interior. Hence the new WMDW crossing the sill became dense enough to cascade down to the bottom of the Tyrrhenian Sea. However, given the higher heat and salt content of the Tyrrhenian resident deep waters (~13 °C, ~38.5, e.g.^22^), compared to the rest of the WMED, this lead to a positive jump in density, but negative jumps in both temperature and salinity in the bottom layer. The consequence is a different stratification, with warmer and saltier water overlying fresher and colder water, a situation prone to salt fingering. This mixing regime is more effective than the double-diffusive convective regime (occurring between old and new deep waters in the rest of the WMED^23^), which might explain why in the Tyrrhenian there is not the characteristic long-lasting sharp interface between old and new deep waters (Fig. 4A vs 4B): here the salt fingers transporting heat and salt downward slowly increase the heat and salt content of the bottom layer, allowing the interface with the layer above to gradually fade away.

### Westward propagation of the anomaly

**Figure 5:**
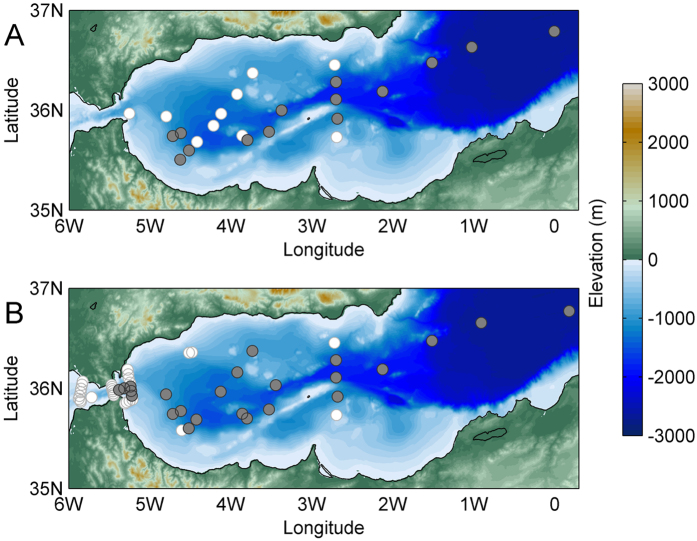
Spreading of the new WMDW (stations in grey) within the Alboran Sea and the Strait of Gibraltar during a survey in (**A**) November 2008 (grey) stations where σ > 29.108 kg m^−3^) and in (**B**) August 2010 (grey stations where σ > 29.11 kg m^−3^) (map generated by using MATLAB 7.1, http://uk.mathworks.com/products/matlab).

On the western edge of the transect sketched in Fig. 1 there is the gateway to the Atlantic Ocean. Early experiments in the Strait of Gibraltar^24^ have demonstrated the existence of a mechanism (called Bernoulli aspiration) by which high speed shallow flows within the strait are capable of sucking deep Mediterranean Water into the adjacent shallow Alboran Sea and then up and over the sill into the Atlantic. After the onset of the WMT the new WMDW has been brought to much shallower depths inside the Alboran by this mechanism. As early as 2008 its interface with the overlying water was found as shallow as 900 m in the Alboran Sea, while in the WMED interior the same isopycnal was located 1 km deeper (Fig. 1). The unequivocal detection of the winter-2004/05 deep water at about 50 km from the Strait of Gibraltar (thanks to the particular shape in the θS diagram and the high density, >29.108 kg m^−3^, i.e. higher than the density of the old WMDW), allows a estimate of the temporal scales of its spreading: a deep water mass formed in February-March 2005^25^ in the northern WMED has nearly reached Gibraltar in 33 months. The route of the WMDW hypothesized by Bryden and Stommel^26^ is confirmed by the 2008 data (Fig. 5A): it flows westward along the Moroccan slope, an indication of the anticyclonic Alboran gyre extending throughout the water column. However, the signature of new WMDW was still too weak to resist the strong mixing at Gibraltar and hence cannot be found at stations further west. In 2010 the signature of the new dense waters (σ was even higher then, >29.11 kg m^−3^) was found within the strait (Fig. 5B) at longitude 5.46 °W (density threshold at 730 m, over a bottom depth of 940 m); 20 km further west no signature could be detected (bottom depth 330 m).

## Discussion

Over 1950–2010, below 1000 m the Mediterranean underwent the strongest salinity gain anywhere in the world ocean^27^. Two processes enabled the warming and salinification to penetrate to such great depths^11^: intermittent deep water formation events, producing increasingly salty and warm waters and a steady downward double-diffusive heat/salt flux between LIW and WMDW (downward black arrows in Fig. 1), where the stratification is prone to salt fingers and staircases are often observed (Fig. 4A). The injection of new warm and salty deep waters near the bottom forms a transition zone where cold and fresh water (the old deep water) overlies warm and salty water (the new deep water). This kind of stratification is prone to double-diffusive convective mixing, which, even if less effectively than the salt fingering acting above, transfers heat and salt upward into the base of the halocline/thermocline (upward black arrows in Fig. 1). Overall, with the onset of the WMT, the result is a continuous thickening of a well mixed warm and salty layer resulting from processes that accumulates heat and salt (from above and from below) into a restricted depth range^11^, a process that is accelerating the trends observed over the past 60 years in the deep WMED.

Recent studies highlighted the importance of the Mediterranean Outflow Water (MOW) properties and volume in modulating the global ocean circulation and climate pattern, being critical for deep water production in the North Atlantic to which it provides salty and warm water^28^,^29^. The recent significant density increase of water masses feeding the MOW might potentially be able to shift its equilibrium depth in the North Atlantic to deeper levels, adding more uncertainty over the extent to which MOW is able to influence the ocean circulation and the global climate^30^.

A number of studies have addressed different aspects of the WMT, but in a rather fragmented fashion (either focusing on a restricted area, temporal interval or process). This study contributes to the understanding of the temporal and spatial evolution of all elements of the enhanced thermohaline variability in the WMED, consisting in a number of features that occurred during the last decade and that have not been previously observed. The filling up of the WMED with new anomalous dense waters, the stepwise warming, salinification, densification and ventilation of deep waters, a new stratification prone to different double diffusive mixing regimes, the perturbation and ventilation of the deep Tyrrhenian and the potential impact on the North Atlantic are all indicators of a broken “equilibrium” that had been in place for decades. Such significant modifications in the thermohaline circulation of the Mediterranean will impact the abyssal ecosystem structure as well as the carbon cycle. The correct simulation of this climate shift will represent a significant challenge to the climate modelling community.

## Methods

The map in Fig. 3 shows the position of more than 1600 CTD stations. Data have been collected during 30 cruises from 2004 to 2015, carried out by the Italian National Research Council (CNR) on board of R/V URANIA (2004–2014) and R/V MINERVAUNO (2015). In all hydrological stations continuous vertical profiles of conductivity, temperature and pressure were obtained from the surface to the bottom by means of a CTD SBE 911 plus system. Temperature measurements were performed with a SBE-3/F thermometer (resolution 10^−3^ °C) and conductivity measurements with a SBE-4 sensor (resolution 3 × 10^−4^ S m^−1^). In addition, salinities of water samples collected in about 1/3 of the station during each cruise were analyzed on board using a Guildline Autosal salinometer. Probes are generally calibrated before and after each cruise, either at the manufacturer or in the calibration facility of CMRE in La Spezia, Italy. The fixed point data collected in the Sardinia Channel come from a deep sea mooring equipped with a near-bottom SBE37 (temperature, conductivity) probe (calibrated each year by the manufacturer), that has been deployed in July 2003 at 1900 m depth (38° 20.05′N, 009° 19.96′E), in the framework of the HYDROCHANGES Programme (CIESM, http://www.ciesm.org/marine/programs/hydrochanges.htm^18^), by the Tunisian Institut National des Sciences et Technologies de la Mer (INSTM).

## Additional Information

**How to cite this article**: Schroeder, K. *et al.* Abrupt climate shift in the Western Mediterranean Sea. *Sci. Rep.* **6**, 23009; doi: 10.1038/srep23009 (2016).
